# Delayed Recovery After General Anesthesia due to Undiagnosed Pseudocholinesterase Deficiency: A Case Report

**DOI:** 10.1155/cria/8426572

**Published:** 2026-09-09

**Authors:** Dochka Tzoneva Tobova, Maryna Uporova, Ivanka Istalianova Dimova, Elena Georgieva Mihaylova

**Affiliations:** ^1^ Clinic of Anesthesiology and Intensive Care, University Hospital of Orthopedics “Prof. B. Boychev”, Sofia 1614, Bulgaria; ^2^ Medical University Sofia, Sofia 1431, Bulgaria, mu-sofia.bg; ^3^ Molecular Medicine Centre, Medical University Sofia, Sofia 1431, Bulgaria, mu-sofia.bg

**Keywords:** case report, delayed recovery, general anesthesia, pseudocholinesterase deficiency, succinylcholine

## Abstract

Pseudocholinesterase deficiency is a rare pharmacogenetic disorder caused by a defect in the enzyme butyrylcholinesterase, which may result in delayed recovery following exposure to neuromuscular blocking agents such as succinylcholine and mivacurium. Patients with this condition require mechanical ventilation and sedation until they regain muscle strength and can be safely extubated. This report highlights the clinical implications of pseudocholinesterase deficiency and discusses the approach to providing safe and effective anesthesia for affected patients. We present the case of a 61‐year‐old woman who underwent elective vertebroplasty for a vertebral compression fracture and experienced delayed recovery from general anesthesia with prolonged postoperative apnea, which was subsequently determined to be caused by hereditary pseudocholinesterase deficiency. In conclusion, early recognition of prolonged neuromuscular blockade after general anesthesia due to pseudocholinesterase deficiency is essential for prevent complications and potentially life‐threatening outcomes.

## 1. Introduction

Pseudocholinesterase (PChE) deficiency is a rare condition that may lead to unexpected perioperative complications and has important implications for patient safety. In affected individuals, the body cannot effectively metabolize choline ester agents, including the neuromuscular blocking agents succinylcholine and mivacurium, which are commonly administered during induction of general anesthesia (GA) to facilitate tracheal intubation, as well as the ester‐type local anesthetics (procaine, chloroprocaine, tetracaine, and cocaine) [1]. As a result, prolonged neuromuscular paralysis and respiratory insufficiency may occur.

Butyrylcholinesterase (BCHE), also referred to as PChE, plasma cholinesterase, serum cholinesterase, or “false” cholinesterase, is a liver‐synthesized enzyme that is widely distributed throughout human tissues, except erythrocytes [1]. PChE deficiency may be either acquired or inherited [1, 2]. Hereditary PChE deficiency is an autosomal recessive condition caused by variants in the *BCHE* gene located on Chromosome 3 (3q26.1‐q26.2) [3]. These variants result in reduced plasma PChE levels, decreased enzymatic activity, or impaired protein stability [2, 4]. The most common pathogenic variants are the A (atypical) and K (Kalow) variants. Acquired causes of PChE deficiency include liver disease, renal disease, malnutrition, pregnancy, advanced age, malignancy, burns, and drug interactions [1, 2]. The duration of residual neuromuscular blockade depends on the degree of enzymatic impairment. Patients with acquired deficiency or those who are heterozygous carriers of a pathogenic variant typically experience prolonged paralysis lasting up to 2 h after succinylcholine administration, compared with the usual duration of 5–12 min (depending on the administered dose) in individuals with normal PChE activity [2, 5]. In contrast, homozygous carriers of pathogenic variants may develop profound and prolonged neuromuscular blockade lasting 4–8 h.

Because PChE deficiency is clinically silent, it is usually recognized only after unexpected prolonged neuromuscular blockade following administration of succinylcholine or mivacurium during induction of GA [2, 3, 6]. Prolonged neuromuscular paralysis due to PChE deficiency may lead to serious physical complications, including apnea, respiratory failure, and the inability to extubate the patient and discontinue mechanical ventilation, as well as psychological sequelae such as posttraumatic stress disorder [7].

PChE deficiency has a favorable prognosis. Currently, no specific antidote is available to treat this condition. Management is primarily supportive and includes close patient monitoring, ventilatory support, and adequate sedation until neuromuscular function has recovered sufficiently to permit safe extubation [3, 7–10]. To increase plasma BCHE activity and potentially shorten recovery time, transfusion of fresh frozen plasma, whole blood, or human serum PChE has been proposed. However, this approach is currently considered unnecessary because of the additional risks and costs associated with transfusions. Furthermore, supportive care with mechanical ventilation and sedation is usually sufficient and remains the standard of care [2, 3, 11].

This report reviews the clinical implications of PChE deficiency and highlights key considerations for safe anesthetic management by presenting the case of delayed recovery from GA caused by previously undiagnosed hereditary PChE deficiency.

## 2. Case Presentation

A 61‐year‐old White woman (weight, 51 kg; body mass index, 19.9 kg/m^2^) with an osteoporotic T8 vertebral compression fracture was scheduled for elective vertebroplasty under GA. Her medical history included tobacco use and two prior emergency laparotomies (the first for blunt abdominal trauma with splenic rupture and the second for adhesive ileus), both performed under GA without reported anesthetic complications. There was no family history of anesthesia‐related adverse events. Preoperative evaluation and routine laboratory tests were unremarkable, and the patient was classified as American Society of Anesthesiologists (ASA) Physical Status II [12]. No causes of acquired PChE deficiency were identified based on the patient’s history [1, 2].

The GA protocol included premedication with famotidine (20 mg intravenously [IV]), metoclopramide (10 mg IV), dexamethasone (4 mg IV) for postoperative nausea and vomiting prophylaxis, and fentanyl (100 μg IV). GA was induced with propofol (200 mg IV) and succinylcholine (100 mg IV) to facilitate tracheal intubation using a 7.0‐mm armored cuffed endotracheal tube. Anesthesia was maintained with sevoflurane (minimum alveolar concentration [MAC], 1) in a 50:50 air–oxygen mixture delivered through a circle breathing system and synchronized intermittent mandatory ventilation using a GE Carestation 650 Anesthesia Delivery System (GE HealthCare, China). No additional neuromuscular blocking agents were administered. Standard basic anesthetic monitoring was performed in accordance with ASA guidelines [13]. Cefazoline (2 g IV) was administered for perioperative antibiotic prophylaxis. At the end of the procedure, acetaminophen (1 g IV) and tramadol (100 mg IV) were administered for postoperative analgesia. The surgery, performed with the patient in the prone position, lasted approximately 30 min and was uneventful. Throughout the procedure, the mean arterial pressure remained above 65 mmHg, and oxygen saturation was maintained at 98%–100%. Additionally, normothermia (body temperature, 36.4°C–36.8°C), normocarbia (end‐tidal CO_2_, 30–34 mmHg), and normoglycemia (blood glucose at the end of surgery, 4.8 mmol/L) were maintained.

Following discontinuation of anesthetic agents, the patient remained apneic and unresponsive for more than 30 min. Blood samples for a complete blood count, blood gas analysis, and biochemical analyses were immediately collected and processed in the hospital laboratory to rule out hypoxemia, hypercarbia, electrolyte disturbances, and hypoglycemia. All results were within the reference range. Administration of naloxone and flumazenil failed to reverse the condition, and metabolic, respiratory, and neurologic causes were excluded. The patient was transferred to the intensive care unit while intubated for continued mechanical ventilation and sedation. Given the prolonged neuromuscular blockade after a single dose of succinylcholine, PChE deficiency was suspected, and one unit of fresh frozen plasma was administered. Spontaneous ventilation and neuromuscular function gradually recovered, allowing extubation 145 min after surgery (190 min after succinylcholine administration). Additionally, a blood sample was obtained to determine BCHE activity and processed at an external laboratory (Ramus Laboratory, Sofia, Bulgaria). The results, received the following day, revealed markedly reduced plasma BCHE activity of 2810 U/L (reference range: 5320–12,920 U/L). The patient did not report any distressing experiences related to the delayed recovery from GA. Subsequently, the remainder of the postoperative course was uneventful, and the patient was discharged on Postoperative Day 3.

Twelve days later, the patient underwent repeat vertebroplasty at the L1 and L3 levels under GA induced with propofol alone. Tracheal intubation was performed without neuromuscular blockade. Recovery was uneventful, and the patient was extubated 10 min after completion of surgery.

We hypothesized that, in this case, the PChE deficiency was likely congenital because the patient was not taking any medications and had no conditions known to cause acquired PChE deficiency [1, 2, 14]. The definitive diagnosis was established by direct sequencing of the *BCHE* (NM_000055.2) gene, which demonstrated heterozygosity for the pathogenic variant c.293A > *G* (p.Asp98Gly; rs1799807) and the hypomorphic variant c.1699G > *A* (p.Ala567Thr; rs1803274). Both variants are associated with reduced BCHE activity and prolonged paralysis following exposure to succinylcholine or mivacurium. The first corresponds to the atypical (A) BCHE variant (dibucaine‐resistant), and the second corresponds to the much milder Kalow (K) BCHE variant (associated with an approximately 30% decrease in enzyme activity). Genetic analysis was performed at the Molecular Medicine Center, Medical University, Sofia, Bulgaria. DNA samples were obtained and stored after the patient provided written informed consent. Parental segregation analysis was not feasible. The patient received genetic counseling and was advised to inform future healthcare providers of her diagnosis and to consider genetic testing for family members. At the time of this publication, the dibucaine number had not been determined.

The patient was informed that her condition was hereditary and was advised to have her children tested. Additionally, she was given clear instructions to alert future anesthesia providers if she or her family members require GA in the future. In accordance with local regulations, the information regarding the congenital PChE deficiency was formally recorded in the patient’s electronic health record. To maximize clinical visibility, this finding was explicitly integrated into the patient’s medical alert and allergy list.

**FIGURE 1:**
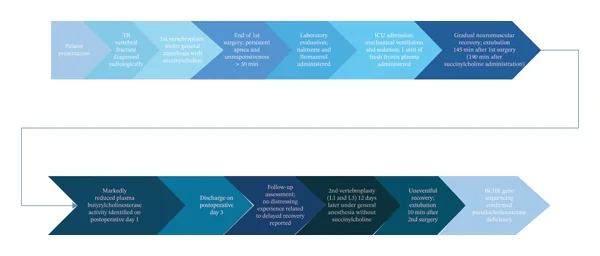
Timeline of the patient’s clinical course. Abbreviations: BCHE = butyrylcholinesterase; ICU = intensive care unit; L1 = first lumbar vertebra; L3 = third lumbar vertebra; min = minutes; T8 = eighth thoracic vertebra.

The patient’s clinical course is presented chronologically in Figure 1.

## 3. Discussion

PChE deficiency (ICD‐10: E88.0) is a rare but clinically important condition associated with GA that manifests as prolonged neuromuscular blockade and respiratory insufficiency. Its incidence is estimated at 1 in 2000–5000 individuals [3], with a higher prevalence in men (2:1) and in certain populations, including White males of European descent, Persian Jews, and subgroups of the Alaska Native population [14].

PChE deficiency is usually clinically silent and becomes apparent in the perioperative period after exposure to triggering agents, manifesting as delayed recovery from anesthesia with prolonged paralysis and apnea. Consequently, anesthesiologists should maintain a high index of suspicion for PChE deficiency in patients with delayed emergence from anesthesia. In patients with known PChE deficiency or a positive family history, succinylcholine and mivacurium should be avoided in favor of alternative neuromuscular blocking agents. Quantitative neuromuscular monitoring (e.g., train‐of‐four) and processed electroencephalographic monitoring should be used to guide recovery, minimize the risk of intraoperative awareness, and ensure safe extubation [7, 8, 10]. If the condition is unrecognized and triggering agents are administered, prolonged postoperative mechanical ventilation, sedation, and intensive care monitoring may be required. Conversely, no specific postoperative measures are necessary when these agents have been avoided.

In patients with delayed recovery and prolonged apnea following GA with succinylcholine or mivacurium administration, other potential causes should be considered. These include opioid overdose, residual neuromuscular blockade, hypoxemia and/or hypercapnia, hypothermia, hypoglycemia, electrolyte abnormalities (hypermagnesemia, hypophosphatemia, and hypokalemia), cerebrovascular accident, cholinergic crisis, myasthenia gravis, and myasthenic syndrome [3, 15, 16]. Prompt identification and correction of the underlying cause are essential [17].

Routine preoperative investigations do not typically detect PChE deficiency. Available diagnostic tests include measurement of plasma BCHE activity and determination of the dibucaine number [9]. Molecular genetic analysis of the *BCHE* gene is considered the gold standard for definitive diagnosis [1, 3, 15, 18, 19].

When planning the patient’s subsequent anesthesia, following confirmation of reduced plasma BCHE activity, we considered two approaches: (1) intravenous induction of GA with the nondepolarizing neuromuscular blocking agent atracurium to facilitate tracheal intubation or (2) intravenous induction of GA with apnea followed by tracheal intubation without the use of neuromuscular blocking agents. We chose the second option because the previous anesthesia had demonstrated uncomplicated tracheal intubation (Cormack–Lehane Grade I), making neuromuscular blockade unnecessary [20].

The absence of anesthesia‐related complications during the patient’s previous surgeries (for blunt abdominal trauma with splenic rupture and for adhesive ileus) may be explained by several factors. We speculate that the prolonged duration of the emergency procedures (likely exceeding 3 h each) and the administration of blood products, including fresh frozen plasma, may have partially restored plasma PChE activity before emergence from anesthesia. Alternatively, postoperative mechanical ventilation may have been continued until spontaneous recovery of neuromuscular function occurred. Given the clinical circumstances, succinylcholine was likely administered as part of rapid‐sequence induction of GA during both emergency procedures. However, because the relevant medical records are unavailable, these explanations cannot be confirmed.

This successfully managed case prompted the addition of neuromuscular monitoring (train‐of‐four) as a mandatory component of basic anesthetic monitoring at our hospital. Since the patient did not report any distressing experiences related to the delayed recovery from GA, further consultations were not considered necessary.

The recommended approach to safe perioperative anesthetic management in patients with PChE deficiency includes the following: (i) obtaining a thorough personal and family history of anesthesia‐related complications; (ii) performing plasma BCHE activity testing and genetic analysis for the patient and, when appropriate, family members; (iii) developing an individualized anesthetic plan that avoids agents metabolized by PChE, including succinylcholine, mivacurium, and ester‐type local anesthetics; (iv) implementing quantitative intraoperative neuromuscular monitoring (e.g., train‐of‐four) together with anesthesia depth monitoring; (v) providing continued mechanical ventilation and adequate sedation until neuromuscular function and spontaneous ventilation are restored; and (vi) ensuring that patients inform all future healthcare providers of their diagnosis before undergoing anesthesia.

The limitations related to the single‐case nature of this report, the limited role of routine preoperative screening for PChE deficiency, and the potential for recall bias regarding previous anesthetic exposures have been acknowledged and discussed. These factors may influence the generalizability and interpretation of the findings; however, the case provides valuable clinical insight into the recognition and management of delayed recovery after GA due to undiagnosed PChE deficiency.

## 4. Conclusion

Early recognition of prolonged neuromuscular blockade after GA due to PChE deficiency is essential for preventing complications and potentially life‐threatening outcomes. Physicians should be familiar with the clinical manifestations, pathophysiology, diagnosis, and management of this condition. Appropriate supportive care and avoidance of triggering agents during future anesthetic procedures are the key to ensuring patient safety.

## Author Contributions

Maryna Uporova and Elena Georgieva Mihaylova managed the patient; Ivanka Istalianova Dimova performed the molecular genetic testing and was involved in editing manuscript; Dochka Tzoneva Tobova was involved in preparing the initial and final manuscript.

## Funding

No funding was received for this manuscript.

## Disclosure

All authors have read and approved the manuscript. The authors declare that the material has not been published before.

## Ethics Statement

Written informed consent was obtained from the patient for the publication of this report, ensuring patient confidentiality.

## Consent

Please see the Ethics Statement.

## Conflicts of Interest

The authors declare no potential conflicts of interest.

## Data Availability

Data are available upon request.
